# Life values as predictors of pain, disability and sick leave among Swedish registered nurses: a longitudinal study

**DOI:** 10.1186/1472-6955-10-17

**Published:** 2011-09-29

**Authors:** Annika Nilsson, Eva Denison, Per Lindberg

**Affiliations:** 1 Department of Public Health and Caring Sciences, Section of Caring Sciences, Uppsala University, Uppsala, Sweden; 2 Department of Health and Caring Sciences, University of Gävle, Gävle, Sweden; 3 Department of Caring and Public Health Sciences, Mälardalen University, Västerås, Sweden; 4 Department of Psychology Uppsala University, Uppsala, Sweden

## Abstract

**Background:**

Prospective studies on high-risk populations, such as subgroups of health care staff, are limited, especially prospective studies among staff not on sick-leave. This paper is a report of a longitudinal study conducted to describe and compare the importance and consistency of life domains among registered nurses (RNs) working in a Swedish hospital and evaluate a model based on the consistency of valued life domains for prediction of pain, disability and sick leave.

**Method:**

Importance and consistency ratings of life values, in 9 domains, were collected during 2003 and 2006 from 196 RNs using the Valued Living Questionnaire (VLQ). Logistic regression analyses were used for prediction of pain, disability and sick leave at the three-year follow-up. The predictors family relations, marriage couples/intimate relations, parenting, friends/social life, work, education, leisure time, psychological well-being, and physical self-care were used at baseline.

**Results:**

RNs rated life values regarding parenting as most important and with the highest consistency both at baseline and at follow-up. No significant differences were found between RNs' ratings of importance and consistency over the three-year period, except for friends/social relations that revealed a significant decrease in importance at follow-up. The explanatory models for pain, disability and sick leave significantly predicted pain and disability at follow-up. The odds of having pain were significantly increased by one consistency rating (psychological well-being), while the odds were significantly decreased by physical self-care. In the model predicting disability, consistency in psychological well-being and education significantly increased the odds of being disabled, while consistency in physical self-care significantly decreased the odds.

**Conclusion:**

The results suggest that there might be a link between intra-individual factors reflecting different aspects of appraised life values and musculoskeletal pain (MSP).

## Background

One professional group at risk for developing pain and disability are registered nurses (RNs) who work in hospital settings, and who are involved in patient care or hold administrative positions. These nurses are subject to physical, psychological and psychosocial demands [1-5], and Hignett reported that more frequent patient handling appears to be related to increased complaints of musculoskeletal pain (MSP) [6]. Such complaints also negatively affect several domains in individuals' daily life [7-9]. As a result, many MSP sufferers will be hindered in their everyday life, unable to do things they like or prefer to do. Thus, individual life values may be challenged when performance of activities or social interaction is hindered [10].

Values have been defined as "a higher order concept thought to provide a structure for organising attitudes" [11] p174 where both values and attitudes often are measured differently. Hyde and Weathington studied possible linkages between personal values and attitudes in the work sphere. They noticed that these values do play a role in people's work according to the fact that these values affect our attitudes, commitment levels and intentions at work [12].

Personal life values are related to an individual's overall value system [12]. These values have been described as a "person's stable, internalized beliefs about how he or she should behave and have the ability to predict how a person will perceive and evaluate environmental stimuli" [13] p 30. Personal life values incorporate sub-domains in which general life values are related to both work and family domains [13,14]. When the individual's own ideas about how to give mutual priority to these areas are not in accordance with reality, this conflict per se is of importance for general dissatisfaction with life, i.e. in the home and work domains [13].

Previous findings suggest that the relative importance of life and work values varies [14]. Individual life value domains involving health, happiness, love, and physical and economic security were rated the highest among managers and workers in different organizations. Similarly, the most important work domains were job interest, responsibility, and fair supervision. Data on life values assessments among people with physical disorders show that chronic illness or disability affects most of the person's life domains [15-19]. For instance, the importance of areas related to health and mobility among persons with different disabilities and attainment scores in these areas are reduced [15]. Women with primary breast cancer rated the domain positive relations as more important than did healthy controls [17]. These studies had descriptive and comparative designs and involved different life domains (e.g., harmony, mobility, positive relations and communication); respondents rated their personal evaluation of the importance of and attainment in these areas [15-19].

Research on risk factors for the development of persistent MSP among RNs has revealed a complex interaction between several physical and psychosocial factors [6,9,20-23]. A previous longitudinal study among the same RNs as in the present study showed that none of the work-related factors (e.g., satisfaction with work-mates and management) had predictive value for pain, disability or sick leave. Personal and individual factors such as age, self-rated health, and sleep quality predicted disability to some degree, while self-ratings of pain, disability and sick leave were related to the outcome over a three-year period [24].

In the present study, life values in several domains were used to understand how such reports could be used in the study of work-related physical problems, the aim being to use life values as predictors of pain, disability and sick leave. Both importance and consistency (whether the RNs have lived according to their values) scores were collected concerning each domain (e.g., family, parenting, social relations). One previous study used personal life values related to different life domains (i.e., family, intimate relations, friends, work, health, and growth or learning) as a predictor of pain in a clinical sample [10]. However, these authors used a cross-sectional design in which the sum of the consistency scores was used as the predictor. Results showed that living in accordance with life values was related to less disability, depression and pain-related fear. In the present study, consistency scores were used as predictors for pain, disability and sick leave, while RNs' importance ratings of each life domain were used as descriptive measures.

The specific aims of the study were to: a) describe and compare the importance and consistency of ten life domains among RNs working in a Swedish hospital over a three-year period, and b) predict pain, disability and sick leave on the basis of personal life values at baseline.

## Method

### Design

A descriptive, correlation study with longitudinal design was used.

### Procedure and participants

Prospective studies on high-risk populations such as subgroups of health care staff are limited, especially prospective studies among RNs not on sick leave and on life values factors. Therefore the study was carried out among RNs recruited from different departments (n = 23; e.g., medical, surgery, obstetrics and gynaecology departments) of a county hospital in the middle of Sweden during spring 2003. This year, 875 persons were employed as RNs and among them 794 (91.0%) were women and 81 (9.0%) men. Nurse administrators at each department were informed about the study and the data collection procedure. RNs were informed about the study by the first author (AN) and 348 RNs were invited to participate in the study during ward meetings at the baseline data collection in 2003. A convenience sample was used and those who agreed to participate were given a questionnaire with a unique code number. Of 348 RNs, 278 (80.0%) completed the questionnaire, 271 women (97.5%), and 7 men (2.5%); mean age 43 years (sd 9.4). About half of the RNs reported pain, at several and multiple pain sites.

A list of RNs who had completed the questionnaire in 2003 was received from the hospital's chief executive secretary before the three-year follow-up was performed. A questionnaire with the same content was mailed to the subjects during spring 2006, two reminders were sent out.

**Figure 1:**
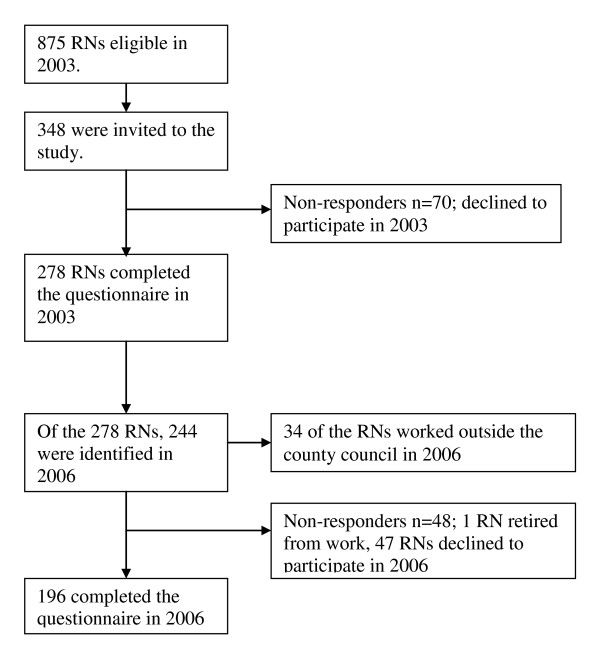
**Participant's response rate and attrition in the years 2003 and 2006 (n = 196)**.

In 2006, 244 (88%) RNs of the original sample were found for the follow-up assessment. Of the 244, 196 (80%) returned the questionnaire (190 women, 97% and 6 men, 3%) Of those who did not participate (n = 82) 34 RNs worked outside the county council in other disciplines, 1 RN was retired from work and 47 RNs declined to participate (see Figure 1).

There were no significant differences between responders and non-responders to the follow-up (2006) questionnaires regarding age, the number of days the person had been on sick leave during the year, years working as an RN, children, marital status or pain problems at the time of the baseline assessment in 2003. During the three-year period, 62 of the RNs (31%) changed departments within the hospital.

### Instruments

Questions concerning self-reported information on each participant's demographic data (gender, age, number of years working at the present job, marital status, present nursing ward, children, present job situation, pain disorders [(yes/no] and sick days during the past year) were posed at baseline 2003. Participants who reported pain disorders were asked to locate their pain (when applicable), pain during the past week (0 to 7 days), days using medication during the past week (0 to 7 days) and perceived limitations caused by pain using a response format of 0 to100 visual analogue scales (VAS; restriction in leisure time). At the three-year follow-up, three questions were added: "Have you changed nursing ward or workplace since 2003 and, if so, for what reason?", "Where do you work today?" and "If you didn't have a pain disorder at the baseline assessment but have one now, how long have you had it?"

Pain, disability and sick leave were measured using three items: number of pain days, perceived limitations caused by pain symptoms in leisure time and being sick listed during the past year. Life values were rated using the Valued Living Questionnaire (VLQ) [25,26]. The VLQ originally presented ten domains (each domain consists of one item): family relations (other than parenting and intimate relations), marriage couples/intimate relations, parenting, friends/social life, work, education, leisure time, psychological well being, citizenship, and physical self-care (health-related issues, e.g., sleep, diet and exercise) [25]. Participants were asked to rate the importance of each domain as well as if they have lived in accordance with their values (i.e. consistency) during the past week. Each domain is estimated on a 10-point scale (1 = no importance/consistency and 10 = great importance/consistency). In the present study, the domain of citizenship was not rated by a majority of the participants. Therefore, these data were excluded from the study. In the original VLQ, Chronbach's alpha for importance was 0.90, and for consistency the value was 0.75 [25]. In the present study, Chronbach's alpha for importance was 0.73, and for consistency 0.77.

### Data analyses

In order to create dependent variables for the logistic regression model, three new categorical variables were constructed on the basis of the original items used in the 2003 and 2006 questionnaires. 1) The number of days rated as pain-free were transformed to a categorical value "pain", (yes/no; 1 to 7 days = yes = 1 and 0 days = no = 0). 2) "Disability" originally concerned perceived limitations caused by pain symptoms during leisure time (0 to 100 VAS) using a cut-off point set at 20 [27]. Subjects reporting a value lower than 20 were not considered disabled, while those scoring 21 to 100 were considered disabled. 3) "Sick leave" was assessed using a single item on the total amount of sick-listing annually. Subjects who were sick-listed for more than seven days were labelled "yes", while those reporting fewer days were labelled "no". The cut-off point is based on the standards of the general insurance system in Sweden, where illness longer than seven days requires a medical certificate from the physician to ensure eligibility for sickness benefits.

All data were analysed using the Statistical Package for the Social Science (SPSS). Data on subject's characteristics and their valued life domains from 2003 and 2006 are presented in terms of frequency, means (standard deviation), range and percentage. Wilcoxon's rank tests were used to analyse differences between 2003 and 2006 for pain disorders, pain sites, sick days (yes/no), disability (< 20 and > 20) [27], and sick leave > 7 days [28]. Dependent t-test of paired samples were used to analyse differences in age, years working as a RN, pain and use of medication during the past week, annual sick leave and valued life domain scores. Missing values were substituted with means for the available data on life domains when the missing values were > 10% [29].

Spearman's test (*r*) was used to explore correlation between the variables. Logistic regression analyses were used for prediction of pain, disability and sick leave at the three-year follow-up. Family relations, marriage couples/intimate relations, parenting, friends/social life, work, education, leisure time, psychological well-being, and physical self-care were used as predictors at baseline. According to Tabachnick and Fidell N should be greater that 50 + 8 times the number of predictors [30]. In this study there were 9 predictors and the number of study participants required would be at least 122 (50+72 = 122 subjects). Multicollinearity (values of *r *> 0.9) was not found in the data [30]. The results are presented as odds ratios with 95% confidence intervals. The level of significance was set at 5% for all statistical tests.

### Ethical considerations

The study was approved by the Ethics Committee at the Medical Faculty, Uppsala University (reference number 02-314). RNs who were asked to participate in the study were informed both orally and in writing that their participation was voluntary and that confidentiality would be assured.

## Results

### RNs' background information

Baseline characteristics of the RNs are presented in Table 1. Among the RNs there were significant differences regarding age (t = 18.66, df = 195, p < 0.001) and years working as a RN (t = 3.46, df = 187, p < 0.001) between baseline and follow-up (age m = 45 SD 9.7; working as a RN m = 15 SD 10.4). No other significant differences were found regarding gender, marital status, children, employment or working time.

**Table 1:** Baseline characteristics of the 196 RNs included in the study 2003.

Variables		2003			2006		
	n	m	(SD)	Range	m	(SD)	Range
Gender							
Female	190						
Male	6						
**Age**	**196**	**43.0**	**(9.4)**	**25-63**	**45.0**	**(9.7)**	**27-65**
**Years working as a RN**	**188**	**13.0**	**(11.1)**	**0.5-39**	**15.0**	**(10.4)**	**3.5-42**
Marital status							
Spouses	161						
Single	35						
Children (yes/no)	155/36						
Children at home (yes/no)	111/49						
Employment							
Permanent job	187						
Nurse substitute	8						
Working time							
Full time	111						
Part time	71						

^a ^= Information missing for some RNs at baseline; **Bold figures **= significant, p < 0.001.

Table 2 shows descriptive data for self-reported pain disorders, pain location, annual sick leave, pain during the past week, days using medication and restriction in leisure time at baseline and at the three-year follow-up. Pain related to the musculoskeletal system was common among the RNs at baseline and follow-up. The most common pain sites were the lower back, shoulders and "other locations". Neck pain and sick days significantly increased over time. Sixty-eight of the RNs experienced pain and 68 did not experience pain at both points in time. Thirty-four had pain at baseline, but no pain at follow-up, and 26 had no pain at baseline, but pain at follow-up (not in Table 2).

**Table 2:** Frequency and/or mean scores regarding RNs' demographic data in 2003 and in 2006 (n = 196).

Variables		2003				2006		
	n	(%) M (SD)		Range	n	(%) M (SD)		Range
Pain problems (yes/no)	94/102	(48/52)			102/94	(52/48)		
**^1^Neck**	**29**	**(15)**			**41**	**(22)**		
^1^Shoulders	44	(22)			49	(25)		
^1^Upper back	19	(10)			23	(12)		
^1^Lower back	45	(23)			46	(23)		
^1^Other pain sites	41	(21)			51	(30)		
Sick days during past year (yes/no)	115/81	(59/41)			108/92	(54/46)		
**Number of sick days**			**5.7 (12.4)**	**0 - 90**			**16.4 (44.1)**	**0 - 354**
^1^Pain during past week	93	(47)	3.9 (2.6)	0 - 7	102	(51)	4.6 (2.3)	0 - 7
^1^Days using medications	92	(46)	1.2 (1.9)	0 - 7	100	(50)	0.5 (0.5)	0 - 7
^1, 2^Restriction in leisure time	91	(49)	27.1(25.1)	0 - 85	101	(51)	32.6 (26.6)	0 - 98

^1^Only RNs who experienced pain answered the questions. VAS scales generally run from 0 to 100;

^2 ^= the scales run from positive to negative; **Bold figures **= significant; neck p < 0.05 and number of sick days p < 0.001

### Descriptive data and differences

Table 3 presents descriptive data on the importance and consistency scores for the life domains at baseline and three-year follow-up. Parenting was the domain rated highest by RNs. No significant differences in the importance and consistency scores were found between the two assessment points except that friends/social life significantly decreased in importance (t-value = 2.02, df = 195, p = 0.045) over the three-year period. No significant differences in importance (*t*-value = 0.527, df = 195, p = 0.59) or consistency total scores (sum) (*t*-value = -0.479, df = 195, p = 0.63) were found between baseline and follow-up.

**Table 3:** Mean (m) and standard deviation (SD) for VLQ and the scales total sum (n = 196).

	2003		2006	
Variables	m (SD) Range		m (SD)	Range
*Importance *				
Family relation	8.4 (2.0)		8.6 (1.6)	
Married	9.4 (1.2)		9.3 (1.5)	
Parenting	9.5 (1.5)		9.5 (1.4)	
**Friends/Social life**	**8.8 (1.2)**		**8.6 (1.4)**	
Work	8.0 (1.6)		7.9 (1.6)	
Education	7.9 (1.6)		7.8 (1.5)	
Leisure	7.9 (1.6)		8.0 (1.6)	
Psychological well-being	8.8 (1.5)		8.7 (1.4)	
Physical self-care	8.8 (1.4)		8.7 (1.4)	
*Sum*	77.4 (7.6)	52-90	77.1 (7.3)	46-90
*Consistency*				
Family relation Married	7.0 (2.7) 7.3 (2.6)		7.4 (2.2) 7.4 (2.6)	
Parenting	8.0 (2.2)		8.4 (1.9)	
Friends/Social life	6.8 (2.3)		6.7 (2.9)	
Work	7.9 (1.9)		7.6 (2.0)	
Education	5.7 (2.7)		5.7 (2.6)	
Leisure	6.3 (2.7)		6.2 (2.5)	
Psychological well-being	6.5 (2.5)		6.5 (2.5)	
Physical self-care	6.9 (2.5)		7.0 (2.3)	
*Sum*	62.2 (12.8)	22 - 90	62.6 (11.8)	17-88

The scale runs from 1 (no importance/consistency) to 10 (great importance/consistency); **Bold figures = **p < 0.05.

### Prediction of pain, disability and sick leave

Three logistic regression analyses were conducted for the 196 RNs on pain, disability and sick leave at follow-up (2006) using the baseline (2003) consistency scores as independent variables at baseline (2003). Bivariate correlations using the consistency scores at baseline and pain, disability and sick leave at follow-up are presented below.

#### Relationships among the variables

Spearman's correlation tests revealed weak to moderate correlations between family relations, marriage couples/intimate relations, parenting, friends/social life, work, education, leisure time, psychological well being, and physical self-care scores at baseline. The variables family and leisure showed the weakest correlation (*r *= 0.14, p < 0.05), whereas the strongest correlation was between the variables leisure and physical self-care (*r *= 0.50, p < 0.01). The correlation patterns for pain, sick leave and disability at follow-up showed that pain was slightly associated with both disability (*r *= 0.32, p < 0.01) and sick leave (*r = *0.15, p < 0.05). Sick leave was also weakly associated with disability (*r *= 0.24, p < 0.01). When comparing significant correlations between baseline and follow-up data, there exists a weak indication that work was negatively correlated with sick leave (*r *= -0.14, p < 0.05) while physical self-care was weakly negatively correlated with pain (*r *= -0.17, p < 0.05).

#### Dependent variable pain

A total of 96 RNs with pain and 100 without pain at follow-up were included in the regression analyses. The full model significantly predicted pain at follow-up (chi-square = 18.13, df = 9, p = 0.034), indicating that the predictors as a set accounted for between 8.8% and 11.8% of the variance in pain. Psychological well-being and physical self-care predicted pain as single items in the model. Thus, for every unit of increased consistency in psychological well-being the odds of having pain at follow-up increased by 20%. For every unit of increased consistency in physical self-care, the odds of having pain at follow-up decreased by 24%. In Table 4 we present the Beta coefficients (B), odds ratios (OR), and confidence intervals (CI) for the prediction of pain, disability and sick leave at follow-up by the variable consistency for the nine life domains at baseline (n = 196; df = 1).

**Table 4:** Beta coefficient (B), odds ratio (OR), and confidence intervals (CI) for the prediction of pain, disability and sick leave at follow-up by variable consistency for nine life domains at baseline (n = 196) df = 1.

Predictors	Pain B OR (95% CI)		Disability B OR (95% CI)		Sick- leave B OR (95%CI)	
		n = 196		n = 196		n = 196
Family relation	- 0.09	0.0.92 (0.81-1.04)	- 0.14	0.87 (0.76-1.00)	- 0.07	0.93 (0.80-1.07)
Married	- 0.06	0.0.94 (0.82-1.08)	0.32	1.03 (0.89-1.20)	0.83	1.09 (0.92-1.28)
Parenting	0.14	1.1.15 (0.98-1.34)	0.42	1.04 (0.88-1.23)	- 0.89	0.92 (0.77-1.09)
Social relations	- 0.03	0.0.97 (0.84-1.13)	- 0.28	0.97 (0.83-1.14)	- 0.11	0.90 (0.76-1.07)
Work	- 0.04	0.0.97 (0.82-1.14)	- 0.16	0.86 (0.72-1.02)	- 0.22	**0.80 **(0.67-0.96)
Education	0.03	1.1.10 (0.97-1.25)	0.14	**1.15 **(1.00-1.32)	0.06	1.07 (0.92-1.23)
Leisure time	0.04	1.1.04 (0.91-1.20)	0.04	1.04 (0.90-1.21)	- 0.03	0.97 (0.83-1.13)
Psychological well-being	0.19	1.**1.20 **(1.03-1.41)	0.23	**1.26 **(1.06-1.50)	0.01	1.03 (0.86-1.20)
Physical self-care	- 0.28	0.**0.76 **(0.64-0.90)	- 0.21	**0.81 **(0.68-0.96)	0.06	1.06 (0.89-1.27)

*Full models *						
Omnibus;chi^2^, (df 9)		**18.13**		**17.63**	10.89	

**Bold figures **= significant values; the predictors scale runs from 1 to 10 where 1 = no consistency and 10 = full consistency; Significant value for; **Pain**: psychological well-being p = 0.020, physical self-care p = 0.001;

**Disability**: education p = 0.042, psychological well-being p = 0.010, physical self-care p = 0.017;

**Sick leave: **work p = 0.017. **Full models**: pain and disability p < 0.05

#### Dependent variable disability

A total of 140 non-disabled RNs and 56 disabled RNs at follow-up were included in the regression analyses. The full model significantly predicted disability (chi-square 17.63, df = 9, p = 0.040) at follow-up. The predictors as a set accounted for between 8.6% and 12.3% of the variance in disability. Education, psychological well-being and physical self-care significantly predicted disability at follow-up as single items in the model. For education, the odds of being disabled increased by 15% for each unit increase in education whiles the corresponding odds for psychological well-being, increased by 26%. For physical self-care, the odds of being disabled decreased by 19% for each one-unit decrease in this variable (see Table 4).

#### Dependent variable sick leave

A total of 152 RNs with a sick leave duration < 7 days and 44 RNs with a sick leave duration > 7 days at follow-up were included in the regression analyses. The full model did not predict sick leave (chi-square 10.89, df = 9, p = 0.283), indicating that the predictors as a set could not explain the variance in sick leave (see Table 4).

## Discussion

The results showed that RNs' life values regarding parenting were rated as most important and had the highest consistency both at baseline and at follow-up. No significant differences were found between RNs' ratings of importance and consistency scores over the three-year period, except for friends/social relations ratings where the importance had significantly decreased at follow-up. In the explanatory models for pain, disability and sick leave, consistency scores were used as predictors. The models significantly predicted pain and disability at follow-up, but not sick leave. The odds of having pain were significantly increased by one consistency rating (psychological well-being), while the odds were significantly decreased by another (physical self-care). In the model predicting disability, consistency in psychological well-being and education significantly increased the odds of being disabled, while consistency in physical self-care significantly decreased the odds. None of the life values related to daily life predicted sick leave at follow-up.

Several studies of life values have used subjects with physical disorders, while the present study included healthy subjects [15-19]. The general picture emerging here indicates that the domains parenting and family are rated as the most important compared to the other domains. Other data on life values have shown that domains such as health, happiness, responsibility and love were the most important personal areas among employees in various organizations, while job interest, responsibility and fair supervision were the most important domains related to work [14]. Other studies have shown that disabled people rate life values of importance for health and mobility function lower, but give high ratings to the importance of close relations with family and significant others [15,17]. One study revealed results similar to ours, showing that family was rated the most important domain among people with persistent pain, but also that this area was the most satisfactory [10].

In the present study, RNs' ratings of importance and consistency were almost the same at the two assessments. However, importance scores in the domain friends/social relations were significantly lower at follow-up, although the difference was slight. No other significant differences were found. Thus, subjects' importance and consistency ratings of most life value domains appear to be a reasonably stable factor that can be used in prospective correlation studies.

The predictive models for pain, disability and sick leave based on the consistency scores in the valued life domains showed that the models used for pain and disability had explanatory value. However, the models for pain and disability only accounted for 11.8% and 12.3% of the variance, respectively. In a previous study the explanatory models for pain and disability included other personal factors as valid predictors of outcome, although the odds were low [24]. Results from the present study suggest that consistency in life values may serve as a complement to analyses of long-term outcomes for pain and disability. The study by McCracken and Yang, regarding personal values in relation to and acceptance of pain, showed that living in accordance with life values predicts change in functioning, i.e., better functioning, independent of acceptance of pain [10].

The correlations between the few dimensions and the dependent variables are limited and therefore difficult to interpret. We have not been able to locate other research reports against which to compare our results. Possible explanations to our results may be that RNs who reported low psychological well-being might be more likely to experience pain. Chronic pain has been shown to be associated with increased incidence of other symptoms such as depression and anxiety; however causal relations are yet to be established [9].

Studies of life values generally list two different life domains, i.e. work and personal life, where personal life usually includes home-life and family items, while the work domain more specifically includes items on work-related areas [14]. Montgomery listed 82 different life values in ten different domains (harmony, positive relations, mobility, involvement, communication, knowledge, responsibility, comfort, religion and health) among disabled and non-disabled persons [15]. Both importance and attainment ratings were used, but not consistency ratings. These studies had descriptive and comparative designs [17-19]. The Valued Living Questionnaire (VLQ) incorporates both importance and consistency scores and covers ten different life domains. It was originally used as a process measure to highlight the discrepancy between the importance and consistency scores. In the present study, importance scores were used descriptively and consistency scores were used as predictive factors. Therefore no comparisons with other findings could be made.

### Study limitations

The present study uses a longitudinal design, which enables prediction of pain disability and sick leave over time. Logistic regression analyses were used when outcomes were dichotomous and when there were no assumptions about the distributions of the predictor variables due to the fact that the responses on the dependent variable are expected to be nonlinear for one or more of the predictors. The analyses have too little statistical power due to that the expected frequencies are small. The low explanatory power of the predictors may suggest that several other variables are more important or that confounders of the association between exposure (life values) and presented outcomes (pain, disability and sick leave) are present. Other variables such as RNs having experienced other life events or changes between the assessments are potential confounders, and it is known that life events and critical life changes are alternative explanations for increased risk of neck/shoulder pain [31]. During the study period, several organizational changes were made in the hospital and a new hospital superintendent was employed in 2006. Also the sample size may have affected the outcomes. Bonferroni corrections were not applied because of low power in the main analysis due to dichotomization of the dependent variables. Consequently, there may be a risk of mass significance, and some caution in interpreting the results is warranted. Regarding if the results may be generalized to RNs in general, this study was limited to one county in Sweden, but at the same time the present RNs would seem to be typical in terms of their pain complaints and sick leave. Consistency ratings did not predict sick leave in our model. Because sick days were self-reported, and because the RNs may have been on sick leave for reasons other than MSP, sick leave may not have been a valid measure in this case. There may also have been bias associated with assessing different domains of life.

## Conclusions

Several factors influence the development or maintenance of MSP and how individuals interact with the environment. Therefore it is important to approach these factors for early identification of persons who may develop MSP and disability. The present findings might support the idea that looking at different life values in different domains may help us understand how intra-individual factors, together with individual and work-related factors, influence health related outcomes. More specifically the present study suggest that there might be a link between intra-individual factors reflecting different aspects of appraised life values related to MSP. For predictive purposes though, the factors that were related to life values in our study - alone or in combination with other, more established ones - can be important when exploring the development process for MSP-related problems.

## Competing interests

The authors declare that they have no competing interests.

## Authors' contributions

AN, PL and ED were responsible for the study conception and design. AN performed the data collection. AN, PL and ED performed the data analysis. AN was responsible for the drafting of the manuscript. PL and ED made critical revisions to the paper for important intellectual content. All authors read and approved the final manuscript.

## Pre-publication history

The pre-publication history for this paper can be accessed here:

http://www.biomedcentral.com/1472-6955/10/17/prepub
